# Diagnosis and Treatment of Idiopathic Premature Ventricular Contractions: A Stepwise Approach Based on the Site of Origin

**DOI:** 10.3390/diagnostics11101840

**Published:** 2021-10-05

**Authors:** Daniele Muser, Massimo Tritto, Marco Valerio Mariani, Antonio Di Monaco, Paolo Compagnucci, Michele Accogli, Roberto De Ponti, Fabrizio Guarracini

**Affiliations:** 1 Cardiothoracic Department, University Hospital, 33100 Udine, Italy; daniele.muser@gmail.com; 2 Electrophysiology and Cardiac Pacing Unit, Humanitas Mater Domini Hospital, 21053 Castellanza, Italy; m.tritto@libero.it; 3 Department of Cardiovascular, Respiratory, Nephrology, Anaesthesiology and Geriatric Sciences, Sapienza University of Rome, 00161 Rome, Italy; marcoval.mariani@gmail.com; 4 Cardiology Department General Regional Hospital F. Miulli, 70021 Acquaviva delle Fonti, Italy; a.dimonaco@gmail.com; 5 Department of Clinical and Experimental Medicine, University of Foggia, 71122 Foggia, Italy; 6 Cardiology and Arrhythmology Clinic, University Hospital Ospedali Riuniti Umberto I-Lancisi-Salesi, 60126 Ancona, Italy; paolocompagnucci1@gmail.com; 7 Cardiology Unit, “Card. G. Panico” Hospital, 73039 Tricase, Italy; accogli.michele@libero.it; 8 Department of Heart and Vessels, Ospedale di Circolo & Macchi Foundation, University of Insubria, 21100 Varese, Italy; roberto.deponti@uninsubria.it; 9 Department of Cardiology, S. Chiara Hospital, 38122 Trento, Italy

**Keywords:** premature ventricular contractions, transcatheter ablation, antiarrhythmic drugs

## Abstract

Premature ventricular contractions in the absence of structural heart disease are among the most common arrhythmias in clinical practice, with well-defined sites of origin in the right and left ventricle. In this review, starting from the electrocardiographic localization of premature ventricular contractions, we investigated the mechanisms, prevalence in the general population, diagnostic work-up, prognosis and treatment of premature ventricular contractions, according to current scientific evidence.

## 1. Introduction

Premature ventricular contractions (PVCs) in the absence of structural heart disease (SHD), or inherited ion channelopathies, are referred to as idiopathic and are among the most common arrhythmias encountered in everyday clinical practice. They have a focal mechanism and usually originate from specific endocardial or epicardial sites, the right and left ventricular outflow tracts (RV/LV-OT) being the most frequent sites of origin (SOO). Even if isolated PVCs are the predominant clinical manifestation, less frequently they can be accompanied by non-sustained ventricular tachycardia (NSVT) or even sustained ventricular tachycardia (VT) with the same ECG morphology. They generally have a favorable prognosis and can be effectively treated with radiofrequency catheter ablation (CA). A careful analysis of ECG features can help to predict the SOO and plan the procedure. This review aims to present an overview on the current approach to PVCs, starting from the twelve-lead ECG analysis to clinical manifestations and prognosis, and therapeutic strategies including CA.

## 2. Prevalence and Mechanism

Idiopathic PVCs originate, in almost 70% of cases, from the right and left ventricular OT, and they account for 10% of all ventricular arrhythmias (VAs) referred for CA [1]. In particular, the RVOT is the most common SOO, harboring 80% of OT-PVCs, whereas up to 20% of OT-PVCs originate from the LVOT and near structures, including the LV summit and intramural foci in the basal interventricular septum [2]. Within the RVOT, its septal or posterior aspect represents the main source of PVC, accounting for three-quarters of RVOT PVCs, while the remaining originate from the RVOT free wall or anterior aspect and the proximal pulmonary artery. Within the LVOT, the most common SOO are the aortic cusps, with a reported prevalence in large series of cases as high as 15% [3]. However, LVOT PVCs may arise from other near structures, such as the aortic-mitral continuity (AMC), the endocardial aspect of the LVOT and the LV summit, with a cumulative prevalence up to 20% [4]. The remaining 30% of PVCs originate from non-OT structures including the left and right papillary muscles (5–15%), mitral annulus (5%), tricuspid annulus (8–10%), left bundle branch fascicles (10%), cardiac crux and moderator band (Table 1) [2,3,4,5].

Sometimes frequent PVCs can present together with NSVT, or even SVT, which is encountered in about one third of the patients [6,7]. Those manifestations are generally benign while malign PVC-induced ventricular fibrillation (VF) is only rarely seen.

In a population-based study including incident cases between 2005 and 2013, Sirichand at al. found an overall age- and sex-adjusted incidence of idiopathic ventricular arrhythmias among individuals ≥18 years of 51.9 per 100,000, with an increasing incidence with aging [6]. Moreover, although the rate of idiopathic VT was similar across sexes, the age-adjusted incidence of symptomatic PVC was higher in females than males (46.2 per 100,000 vs. 20.5 per 100,000, *p* < 0.001) [6]. In an analysis of gender and age differences in patients undergoing CA of PVCs, the RVOT SOO was 1.5 times more frequent in women than men, while LVOT-PVCs were more common in men and their prevalence increased with aging, as compared to RVOT-idiopathic Vas [7]. Taken together, these observational studies suggest that RVOT and PVCs may be the most frequent SOO and clinical arrhythmia in females.

Frequent PVCs may sometimes lead to PVCs-induced cardiomyopathy (CMP), which is characterized by otherwise unexplained progressive LV dysfunction and heart failure [3,6,8,9,10]. 

From a mechanistic perspective, idiopathic PVCs are focal arrhythmias related to delayed afterdepolarizations (DADs) and triggered activity during phase 4 of action potential [11]. These arrhythmias are usually adrenergically mediated, so that sinus tachycardia facilitates their initiation and are frequently triggered by stress or exertion. The adrenergic stimulus leads to an increased adenylyl-cyclase activity with increased levels of intracellular cyclic adenosine monophosphate (cAMP). cAMP activates the cAMP-dependent protein kinase (protein kinase A, PKA) that phosphorylates the L-type sarcolemmal calcium channels, ryanodine receptor (RyR2) and phospholamban. All these processes lead to increased intracellular calcium concentrations by spontaneous diastolic calcium release from the sarcoplasmic reticulum, known as calcium sparks [11]. Eventually, through the activation of electrogenic sodium–calcium exchanger, a transient sodium inward current enters the cell and produces a DAD, which, if repetitive, may generate VT. This mechanism explains some peculiar characteristics of idiopathic VAs, such as termination by adenosine by lowering cAMP in the ventricular myocardium via an inhibitory G-protein cascade. 

Considered the cAMP-mediated mechanism, it is comprehensible that the pharmacological therapy of idiopathic PVCs is dependent on agents or maneuvers that reduce cAMP levels. Examples include activation of the M2 muscarinic receptor with vagal maneuvers, calcium channel blockers, β-blockers and adenosine (through the activation of the A1-adenosine receptor) [12]. 

## 3. Twelve Leads Electrocardiographic Localization of Premature Ventricular Contractions 

**Figure 1:**
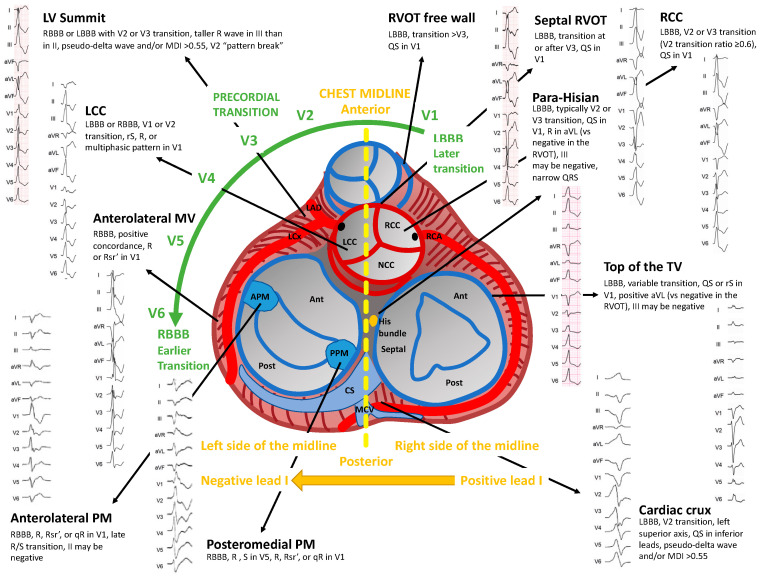
Schematic representation of the main sites of origin of idiopathic premature ventricular contractions and their ECG features.

Twelve-leads surface ECG characteristics, such as frontal plane axis, bundle branch block pattern, precordial transition and QRS width, can be used to predict the most likely SOO. Knowledge of the 3D-anatomy of the heart, its orientation within the chest and the relationship between different cardiac structures is crucial to understand ECG findings. However, it has always to be kept in mind that general rules may have significant variations related to body type, lead placement and relative orientation of the heart to the chest wall. A schematic representation of principal SOO of idiopathic PVCs and their ECG characteristics is presented in Figure 1. 

### 3.1. Outflow Tract Structures 

The RVOT and LVOT are anatomically close, as such, PVCs arising from these structures share some common ECG findings. Being located at the base of the heart (superior within the chest) they all present with an inferiorly directed axis (positive in leads II and III and negative in lead aVL and aVR) and they typically present a left bundle branch block (LBBB) pattern in V1 with various precordial transition according to the specific SOO. The proximal part of the RVOT begins at the superior margin of the tricuspid valve annulus (TVA) and lies to the right of the LVOT, then the RVOT wraps around and crosses the LVOT lying leftward and anteriorly to the aortic root so that the pulmonary valve lies anteriorly, superiorly and to the left of the aortic valve, making the RVOT free wall the most leftward and anterior outflow tract structure. As V1 is a unipolar lead, structures closer to the chest wall show a LBBB pattern with a QS complex, while more posterior structures show a progressive increase in the initial r wave amplitude through a right bundle branch block (RBBB) pattern. As such, when the precordial transition is ≥V4 the PVC are likely to originate from the RVOT. In particular, when the SOO is the RVOT free wall, the precordial transition is typically very late (V4–V5); as the SOO moves progressively more posterior and inferior to the RVOT septum, right coronary cusp (RCC), left coronary cusp (LCC), AMC and lateral mitral valve annulus (MVA), the precordial transition becomes progressively earlier till the V1 pattern transforms to a RBBB pattern. Consistently, there is also a progressive change in the polarity of lead I from negative to positive, moving from structures located leftwards to the chest midline, such as the most anterior part of the RVOT, the LCC, the AMC and the lateral MVA (negative lead I), to structures located to the right of the midline such as septal RVOT and the RCC (positive in lead I). The RVOT free wall and septum extend both leftward and rightward as they curve around the aortic root, thus PVC arising from either the anterior and septal aspect of the RVOT may appear positive, negative or biphasic in lead I. The RCC/LCC junction is typically very close to the midline and therefore may have either a positive, a negative, or a biphasic QRS complex in lead I. When the precordial transition is ≤V2, the SOO is very likely in the LVOT. The RCC is in close proximity to the mid-septal RVOT, thus making it very difficult to differentiate PVC coming from these structures. In general, the precordial transition in V3 represents the most difficult scenario to differentiate RVOT from LVOT PVC, and different algorithms have been proposed [13,14,15,16,17]. A PVC transition later than the transition of sinus rhythm QRS complex is highly specific for an RVOT origin. A V2 transition ratio (defined as the percentage of R wave during the PVC divided by the percentage of R wave during sinus rhythm) ≥ 0.6, instead, predicts an LVOT origin with a high degree of sensitivity and specificity [13]. A V2S/V3R index (defined as the S-wave amplitude in lead V2 divided by the R-wave amplitude in lead V3) ≤ 1.5 is also able to predict an LVOT origin with high accuracy [14]. Compared to PVC coming from the RCC, those coming from the LCC, typically have a significant r wave in V1 due to the more posterior location of the LCC, while RVOT and RCC PVC typically have a QS pattern in V1. Arrhythmias arising from the LCC may also have a multiphasic “M” or “W” pattern in V1 while a QS pattern with notching in downstroke is suggestive of RCC/LCC junction [18,19]. A qR pattern in lead V1 is often seen in VAs from the AMC while a RBBB pattern in lead V1 with positive precordial concordance is suggestive of anterolateral MVA [20].

### 3.2. Left Ventricular Summit and Intramural Left Ventricular Outflow Tract

The left ventricular summit (LVS) is the most superior aspect of the left ventricular ostium, delimited on its epicardial surface by the bifurcation of the left anterior descending (LAD) and the left circumflex (LCx) coronary arteries and transected by the great cardiac vein (GCV) at its junction with the anterior interventricular vein (AIV) [21]. The GCV divides the region into two main areas of clinical interest: (1) a medial and superior region (above GCV), corresponding to the apex of the LVS, inaccessible to catheter ablation (CA) because of its close proximity to the major coronary vessels (inaccessible area); and (2) a lateral and inferior region (below GCV), which may be suitable for CA (accessible area). 

Ventricular arrhythmias originating from the LVS typically show a RBBB pattern with a positive concordance throughout the precordial leads or a LBBB pattern with very early precordial transition (≤V2). The axis is typically rightward and inferior. A dominant R wave in V_1_ with a R/S ratio ≥ 2.5 is observed in up to 90% of cases and predicts an origin from the accessible area [22]. Only PVC from the inaccessible area may present an LBBB pattern [23]. A peculiar pattern is the “pattern break” in V_2_, characterized by an abrupt loss of R wave in lead V_2_ compared to V_1_ and V_3_, suggesting an origin from the anterior interventricular sulcus, which is located opposite to the unipolar lead V_2_, usually in close proximity to the proximal LAD before the take-off of the first septal perforator branch [24]. An epicardial origin is suspected when there is a slurring of the initial portion of the QRS complex, reflecting delayed initial activation of the LV epicardium, which can be quantified as (1) time to earliest rapid deflection in precordial leads (pseudo-delta wave) ≥ 34 ms; (2) interval to peak of R wave in lead V_2_ (intrinsicoid deflection time) ≥ 85 ms; (3) shortest interval to maximal positive or negative deflection divided by QRS duration (maximum deflection index) ≥ 0.55; and (4) time to earliest QRS nadir in precordial leads (shortest RS complex) ≥ 121 ms [24,25,26,27].

### 3.3. Cardiac Crux 

The cardiac crux is an epicardial region at the intersection of the atrioventricular groove and the posterior interventricular groove near the junction of the coronary sinus with the middle cardiac vein. Arrhythmias arising from this region typically have LBBB with an early (V2) transition and a left superior axis with deep QS waves in the inferior leads. They also present one or more of the aforementioned features, suggesting an epicardial origin. The presence of a greater S wave than R wave in V6 is highly specific of an origin from the cardiac crux as the depolarization propagates epicardially from the crux to the apex first, where it enters the Purkinje system in the endocardium and, thereafter, rapidly moves away from V6 towards the base [28].

### 3.4. Mitral and Tricuspid Valve Annuli 

Arrhythmias originating from the mitral valve (MV) annulus present with a RBBB pattern and positive concordance throughout the precordium. The QRS axis is right inferior in anterolateral MV PVCs with a negative QRS complex in leads I and aVL, while lateral and inferolateral MA PVCs may exhibit a right superior axis with negative QRS complexes in the inferior leads and positive in aVL. Sometimes, PVCs arising from the inferolateral MV may exhibit an inferior lead discordance with negative II and positive III. A notching in the downstroke of the Q wave or upstroke of the R wave in inferior leads can be seen in the case of PVCs coming from the free wall of the mitral annulus, representing the late activation of the RV [29].

All PVCs originating from the tricuspid valve (TV) annulus are characterized by a LBBB pattern. Those coming from the lateral TV annulus present a late transition >V3 and a left intermediate axis with a dominant R wave in lead I, a positive deflection in aVL and a positive II/ negative III inferior lead discordance. Notching in the inferior leads can be seen as a result of delayed LV activation. Arrhythmias originating from the septal aspect of the TV annulus, show, instead, an earlier transition in V3 and a narrower QRS duration. The majority of PVCs arising from the septal portion of the TV annulus, present a QS in V1, while most PVCs from the free wall portion exhibit an rS pattern [30].

### 3.5. Para-Hisian 

The main characteristic of para-hisian PVCs is the narrow QRS duration (typically < 130 ms) related to the involvement of the conduction system. Para-hisian PVCs may be mapped from all the structures near to the His bundle region, including the LV septum below the membranous septum, the RCC and the NCC. Generally, they show an LBBB pattern with QS in V1 and a left inferior axis with a dominant R wave in lead I. A peculiar characteristic is the presence of a positive deflection in aVL related to the more rightward and inferior location, compared to the RVOT and RCC. For the same reason lead III can be isoelectric or negative and, generally, there is a III/II R wave ratio < 1. Infrequently, para-hisian PVCs from below the membranous septum may present an RBBB pattern [31].

### 3.6. Left and Right Ventricular Papillary Muscles, Moderator Band and Left Bundle Branch Fasciculi

Arrhythmias originating from the papillary muscles are characterized by a RBBB pattern with a dominant R wave in V1, a late transition (V3–V5) and a wider QRS (median QRS width of 150 ms). Those originating from the APM have a right inferior axis and sometimes an inferior lead discordance with a negative QRS complex in lead II and a positive one in lead III; while those originating from the posteromedial papillary muscle (PPM) have a left superior axis [32,33].

Fascicular PVCs are characterized instead by a narrower QRS complex (<130 ms), an rsR’ pattern in V1 resembling a typical RBBB and an initial q wave in lead I which are almost never seen in papillary muscle arrhythmias. Left anterior fascicle PVCs have a right inferior (left posterior fascicular block pattern), while those originating from the posterior fascicle have a left superior axis (left anterior fascicular block pattern).

Arrhythmias originating from anterior and posterior RV papillary muscles, as well as those originating from the moderator band, all show an LBBB pattern with late transition (>V4) and a left superior axis [34].

## 4. Diagnostic Work-Up

Initial patient evaluation should include detailed clinical history with focus on inherited arrhythmic syndromes, cardiomyopathies and familiar history of SCD, adrenergic substances consumption and metabolic disorders such as hyperthyroidism. Beyond the prediction of the SOO, resting ECG may rise the suspicion of underlying SHD in presence of depolarization or repolarization abnormalities including q waves, QRS fragmentation and inverted T waves. Exercise stress test should always be part of the initial diagnostic work-up, as exercise-induced PVCs or induction of SVT, as well as frequent PVCs occurring during the recovery phase are all markers of increased risk, even in the absence of myocardial ischemia [35,36]. Although PVCs commonly occur in subjects with morphologically normal hearts, it is crucial to exclude an underlying SHD due to its impact on the therapeutic approach and risk stratification. In this regard, cardiac imaging plays a central role (Table 2) [37,38].

**Figure 2:**
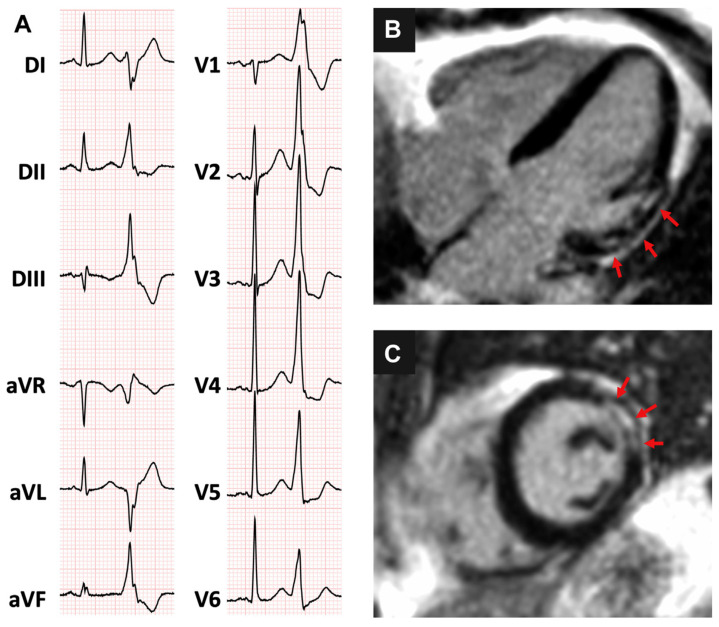
(**A**) 36-year old woman presenting with frequent premature ventricular contractions, with right bundle branch block inferior axis morphology and (**B**,**C**) evidence on cardiac magnetic resonance of a patchy area of subepicardial late gadolinium enhancement, involving the basal anterolateral left ventricular segment (arrows). Reproduced with permission from Muser et al. [50]. Copyright 2020 Elsevier.

Transthoracic echocardiogram (TTE) represents the first line diagnostic test, and its main role is to detect a reduced left ventricular ejection fraction (LVEF), which may either point to an underlying SHD or be secondary to the high PVCs burden (as in PVCs-CMP) [38,39]. LVEF is best measured in the sinus beat after the first post-extrasystolic beat or, in case of bigeminy, by averaging measures taken during PVCs and sinus beats [40,41]. Besides LVEF, echocardiographic assessment should focus on PVCs’ presumed SOO, especially is case of a suspected arrhythmogenic right ventricular cardiomyopathy (ARVC): the presence of right ventricular wall motion abnormalities (akinesia, dyskinesia, aneurysm, bulging), together with a disproportionate RVOT dilation, represent diagnostic criteria for ARVC, and help to differentiate it from training-induced RV re-modeling, which is commonly encountered in athletes [42,43,44]. In case of reduced LVEF, symptoms, cardiovascular risk factors, or other elements suggestive of ischemic heart disease (i.e., presence of abnormal q waves, repolarization abnormalities, regional wall motion abnormalities) invasive or computed tomographic (CT), coronary angiography should be considered to rule out a significant coronary artery disease, with the latter reserved for younger patients, with a lower pre-test probability [45,46]. Cardiac magnetic resonance (CMR) is currently the cornerstone for the assessment of cardiac structure and function, as well as for myocardial tissue characterization. Concealed myocardial structural abnormalities have been reported in up to 50% of patients with unremarkable ECG and echocardiographic findings [47,48,49,50]. Generally, CMR is best reserved for patients with PVCs not arising from the RVOT (i.e., RBBB pattern with superior axis), in presence of multifocal PVCs, exercise induced PVCs, family history of CMP or SCD, older age or when LVEF is reduced (Figure 2) [47,49,51,52].

The main advantage of CMR as compared to TTE lies in the unlimited number of imaging planes, which allows optimal assessment of complex three-dimensional structures, such as the RV [53]. Furthermore, CMR offers non-invasive myocardial characterization capabilities, enabling the detection of fatty infiltration, fibrosis and myocardial edema, which are key elements of the substrate underpinning PVCs in SHD. In addition to diagnostic purposes, the identification and localization of myocardial fibrosis is also important for proper planning of CA and carries significant prognostic implications as the presence of CMR abnormalities has been correlated with increased risk of malignant arrhythmic events during long-term follow-up [50,54,55]. Nonetheless, CMR carries some limitations in patients with frequent PVCs, including gating difficulties and motion artifacts due to the irregular rhythm [56,57]. Furthermore, identification of fibro-fatty replacement of the right ventricular wall may be problematic due to its thin structure [43]. Besides non-invasive diagnostic modalities, electroanatomical mapping (EAM), which is pivotal during CA procedures as it allows the precise localization of the arrhythmic focus by activation mapping, may also provide important diagnostic and prognostic information [58,59]. Notably, EAM allows a very accurate characterization of the myocardial substrate, identifying the presence of abnormal myocardium (i.e., areas of scar or inflammation) as low voltage areas. Some data indicate that EAM has a higher sensitivity than CMR for the detection of myocardial structural abnormalities, even when they have a limited extent such as in early stages of ARVC [60,61]. Furthermore, EAM may serve as a preliminary step for endomyocardial biopsy (EMB), by disclosing diseased myocardial regions in many conditions characterized by a patchy myocardial involvement (i.e., myocarditis, ARVC, sarcoidosis), thus allowing the direct sampling of the diseased myocardium and enhancing EMB’s diagnostic yield [62,63]. The main limitation of EAM lies in its operator dependency, and in the importance of ensuring an adequate tissue contact to avoid the spurious detection of low-voltage areas; in this regard, the introduction of contact-force sensing catheters helped to increase EAM’s accuracy, and to facilitate its standardization [62].

## 5. Prognosis

**Figure 3:**
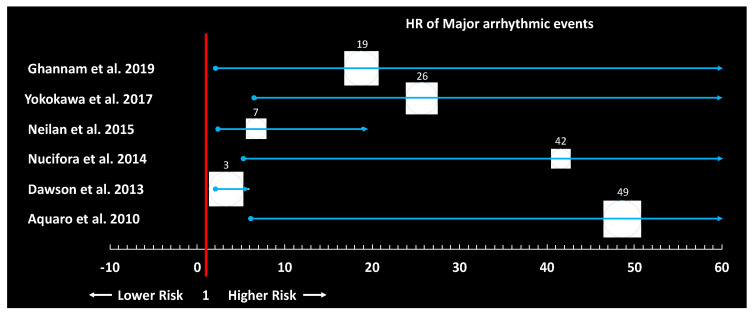
Forest plot showing the results of the principal studies investigating the prognostic role of cardiac magnetic resonance abnormalities in patients with frequent premature ventricular contractions.

The available evidence on the prognostic impact of idiopathic PVCs in terms of risk of death and heart failure is conflicting. Some studies have reported the prognosis of asymptomatic patients with frequent PVCs superimposable to that of the general population, while others have found an increased risk of heart failure and death including SCD [64,65,66]. Two recent large metanalysis, including 11 and 8 large population-based studies and almost 150,000 healthy subjects, have found the presence of PVCs (defined as any PVC occurring ≥1 time during a standard ECG recording or ≥30 times over a 1-h recording and as any PVC documented by a ≥12 s ECG recording, respectively) associated with a 1.7-fold increase in the risk of major adverse cardiac events (MACE) and a 2.64-fold increase in the risk of SCD [67,68]. These data should be carefully interpreted as the major prognostic element in patients presenting with PVCs is represented by the presence of underlying SHD, and the criteria used to define normal heart in the vast majority of the included studies were simply the absence of clinical history of heart disease, normal physical examination and normal resting ECG, with a single study out of 19 having included the use of TTE. More recent data have demonstrated that advanced imaging techniques such as CMR may identify concealed SHD in a non-negligible proportion of patients presenting with apparently idiopathic PVCs, on the basis of a normal resting ECG and TTE [50,53]. Several studies have consistently reported an increased risk of adverse events when CMR abnormalities are detected even in presence of normal LVEF (Figure 3) [50,54,55,69].

On the other hand, a prospective study including 239 patients with frequent RVOT/LVOT PVCs and normal CMR did not show any MACE during a median follow-up of 5.6 years [70]. When CMR abnormalities are detected, further refinement of risk stratification should be considered, especially in patients with preserved LVEF. In this regard, induction of SVT by programmed electrical stimulation has been associated with a significant increased risk of malignant VA, compared to patients with CMR abnormalities which are non-inducible [69].

Enthusiasm around the “PVCs hypothesis”, postulating that PVCs suppression with antiarrhythmic drugs (AAD) could lead to a reduced risk of SCD among patients with recent myocardial infarction and asymptomatic PVCs, was suddenly stopped by the publication of the Cardiac Arrhythmia Suppression Trial (CAST), in which a paradoxical increase in the risk of death was reported in subjects receiving class IC AAD [71]. However, several lines of evidence have subsequently supported the notion that PVC suppression with either AAD or CA may result in improved EF and heart failure symptoms, and these treatments should be considered among patients at higher risk of developing PVC-CMP, especially in cases of high PVC burden. The PVC burden threshold at which AAD and/or CA should be considered is variably defined as >10% (the threshold after which most cases of PVC-CMP occur), >16–24% (the statistically optimal discriminatory cut-off value for PVC-CMP), or >6% (the threshold indicating a potential benefit of CA) [38,41,66,72]. Apart from the PVC burden, other predictors of PVC-CMP include wider PVC QRS duration and epicardial origin of PVCs as a result of a higher degree of LV dyssynchrony during the PVC beat. Interpolated PVCs and PVCs with variable coupling interval have been also associated with a higher risk of PVC-CMP [38,42].

Finally, a history of syncope in a patient presenting with PVCs and a structurally normal heart should be considered a red-flag, possibly pointing to a PVC-triggered ventricular fibrillation/polymorphic ventricular tachycardia and requires a careful assessment [73].

## 6. Medical Therapy and Catheter Ablation

No treatment other than reassurance is needed in patients with PVC without underlying heart disease, or inherited arrhythmogenic disorder, who are asymptomatic or mildly symptomatic [74].

In patients with symptomatic PVCs, β-blockers or non-dihydropyridine calcium channel blockers are considered the first-line treatments [45]. These drugs have a long track record of safety in structurally normal hearts, and β-blockers are useful in patients with coronary artery disease or reduced LV function [45].

β-blockers can decrease the arrhythmic burden and improve symptoms and are particularly effective for sympathetically mediated PVCs. In randomized controlled trials the use of β-blockers resulted in a clinically significant reduction in OT-PVCs and symptoms improvement by reducing the increase in contractility of the post PVC sinus beat [75]. Similarly, non-dihydropyridine calcium channel blockers have demonstrated to be effective in treating OT-PVCs and are considered particularly useful for fascicular VA [76]. For these reasons, in patients with a structurally normal heart, it is reasonable to try a calcium channel blocker if a β-blocker fails (and vice versa). Beta-blockers and calcium channel blockers should be used at the lowest effective dose to relieve symptoms and minimize side effects. The exception to this is patients with a prior myocardial infarction or heart failure; then, doses should be titrated to the maximal tolerated. Failure of a drug may occur because of either non-responsiveness or intolerance. With either type of drug, patients may experience fatigue, hypotension, bradycardia or presyncope. β-blockers may also cause depression and erectile dysfunction, and non-dihydropyridine calcium channel blockers may result in gastrointestinal side effects, such as gastroesophageal reflux and constipation, and can cause leg swelling [45].

For patients who have symptomatic PVCs that are unresponsive to a beta-blocker or calcium channel blocker, or in whom those drugs are poorly tolerated and are not good candidates for CA (because of frailty or multifocal PVCs), treatment with additional AAD such as flecainide, propafenone, sotalol and amiodarone may be considered to reduce the frequency of PVCs and improve symptoms [45,77,78,79,80]. Mexiletine is rarely used as its effectiveness is inferior to either other AAD or CA [79].

Class IC AAD (flecainide and propafenone) are generally well-tolerated and highly effective [77,78,79].These drugs are contraindicated in the presence of coronary artery disease, severe left ventricular hypertrophy, or heart failure. Since the CAST demonstrated an excess of mortality related to flecainide use in an attempt to suppress post myocardial infarction PVCs, class IC AAD became contraindicated in SHD due to their propensity to facilitate re-entrant ventricular arrhythmias [71].

A number of studies have assessed the efficacy of sotalol for suppressing PVCs, particularly in the presence of coronary artery disease. Sotalol is effective for reducing PVC burden; however, it is associated with QT prolongation and torsades de pointes, a risk that must be balanced with its efficacy for PVC suppression [80].

Amiodarone is highly effective and is one of the few AAD that can be safely administered in patients with severely reduced systolic function; however, side-effects associated with its long-term use make it substantially less preferable, especially in younger patients [72].

For patients with PVC-induced cardiomyopathy, amiodarone is reasonable to reduce the PVC burden, improve symptoms and left ventricular function [72]. Class IC AAD have also been shown to be effective for PVC suppression and improving left ventricular function in patients with PVC-induced cardiomyopathy [81]. However, in the last two decades, with progressive improvement in mapping techniques and CA outcomes, CA has become a first-line therapeutic option especially, when a PVC CMP is suspected [82].

The recent expert consensus statement on catheter ablation of VAs reports some recommendations for CA depending on the specific SOO of PVCs, considering its impact on the choice of the initial approach between antiarrhythmic therapy and CA. This consensus also states that patients with PVC who have characteristics that could lead to tachycardia induced cardiomyopathy should be followed up with careful structured clinical follow-up [82].

In a clinical scenario when it is suspected that a high PVCs burden (>15–25%) may play a significant role in LV dysfunction, CA can help to improve LVEF [68,69]. In patients who were non responders to cardiac resynchronization therapy (CRT) with a PVC burden > 22%, Lakkireddy et al. demonstrated that CA of PVCs improved the efficacy of CRT and consequently LVEF together with New York Heart Association (NYHA) functional class [83].

Medical therapy should be considered as first-line therapy in patients in whom ablation is more complex and leads to a higher risk of procedural complications, but in general, CA has a strong recommendation in symptomatic patients who do not tolerate or do not prefer long-term AAD [82,84].

The planning of the ablative procedure starts with the identification of the possible SOO by careful evaluation of the 12-lead ECG, and this specific approach must be tailored taking into account the anatomical structures that are in close proximity and susceptible to injury. Activation mapping and pace mapping are the standard methods used to define arrhythmia origin. Occasionally, activation mapping during spontaneous arrhythmias is limited by infrequent PVCs. In these cases, VA induction may be attempted with isoproterenol infusion and ventricular or atrial burst pacing. Radiofrequency CA should be performed at the site where earliest activation (≥20 ms) is recorded and, ideally, where the pace-map is also optimal (i.e., 12/12 leads) [82].

In case of OT PVCs, the RVOT is mapped first in patients presenting with LBBB and transition ≥ V3, while the aortic cusps and the LVOT is mapped first in cases presenting with RBBB or LBBB with an early transition (≤V2) [82]. When local activation times at multiple adjacent sites (i.e., GCV/AIC, LCC, LV endocardium and RVOT in cases of OT PVCs) have similar values, especially in the presence of suboptimal pacemaps, an intramural origin of the arrhythmic focus should be suspected. For patients with intramural PVCs, standard unipolar RF ablation may not be successful in eliminating the arrhythmias, even if sequentially delivered from multiple adjacent sites. In these cases, bipolar RF ablation, simultaneous unipolar RF ablation or the use of half-normal saline/non-ionic irrigants have been shown to enhance success. Coronary angiography should be performed before ablation from the great cardiac vein, epicardium and in select cases of ablation within the aortic cusps and radiofrequency energy delivery should be deferred if the site is in close proximity (within 5 mm) to a major coronary artery [82].

In case of RV OT PVCs, CA success rates are reported between 80–95%, with a low complication rate [3,78,85]. Moreover, in symptomatic patients with frequent PVCs from the RVOT, CA has demonstrated a higher rate of efficacy compared to medical therapy with either metoprolol or propafenone in a randomized controlled trial. This study enrolled 330 patients with PVCs from RVOT showing that during the one-year follow-up period, PVCs recurrence was significantly lower in patients randomized to CA (19.4%) vs. medical therapy (88.6%). In this scenario, the expert consensus statement favors CA as a first line approach. However, some patients with PVCs from the RVOT and minimal or tolerable symptoms might prefer medical therapy or no therapy [84].

In patients with recurrent ventricular fibrillation triggered by PVCs often the SOO lies in the Purkinije network. In such cases, CA is a standardized approach to avoid further malignant arrhythmic events [86].

Acute suppression of non-OT idiopathic PVCs with RV origin is over 90% (RV papillary muscles, tricuspid annulus and moderator band) but the risk of recurrence especially of PVCs from the parietal band is higher with the need for redo procedures [5,34,87,88]. Compared to PVCs originating from the RVOT, ablation of PVCs originating from the LVOT is more complex and can involve greater procedural risk due to nearby anatomical structures such as coronary arteries or aortic valve cusps [89].

Left ventricular non-OT PVCs have several well-defined SOO, including papillary muscles, MVA and LV summit.

In PVCs originating from intracavitary structures such as papillary muscles and moderator band, the complex anatomy, its variability and the motion during the cardiac cycle, make CA extremely challenging. In these cases, the use of intracardiac echocardiography (ICE) is pivotal to allow real-time visualization and ensure proper catheter contact. Cryoablation may also be an option to improve catheter stability [82].

In particular ablative treatment of PVCs originating from the papillary muscles requires greater catheter stability, with the need for frequent use of the intracardiac echo and a higher risk of recurrence with the necessity of redo procedures in about 30% of the cases [90,91].

In cases of para-Hisian PVCs, cryoablation has been described as an option when radiofrequency CA is deemed to be at high risk of collateral injury of the conduction system or resulted to be ineffective [92].

A subxiphoid percutaneous epicardial approach may be pursued when an epicardial origin is suspected on the basis of 12-lead ECG or after failure of an endocardial approach [93].

## 7. Future Perspectives

Although basic and clinical research is constantly evolving, many aspects of diagnosis and management of PVCs remain unknown. In particular, the identification of patients at risk to develop PVC induced cardiomyopathy or malignant arrhythmic events at follow-up is still largely debated, as well as the identification of patients who may benefit from an advanced diagnostic workup, including CMR.

In terms of invasive management, even if current careful procedural planning based on the twelve-lead morphology of PVCs and imaging evaluation has improved the accuracy of identification of the SOO, and advanced technologies such as ICE or cryoablation have improved procedural outcomes, the best approach for PVCs originating from complex anatomical structures such as papillary muscles and intramural foci remains to be defined.

## 8. Conclusions

Idiopathic PVCs are among the most common ventricular arrhythmias in clinical practice. They originate from well-defined and standardized sites of origin from the right and left ventricle that can be predicted on the basis of specific twelve-leads ECG characteristics [94]. In the absence of underlying structural heart disease PVCs have a good long-term prognosis. In case of a transcatheter ablative treatment, an overall evaluation of the patient is essential, starting from the morphology of the arrhythmia ECG to plan the most effective approach.

## Figures and Tables

**Table 1:** Idiopathic ventricular arrhythmia prevalence, procedural success and risk of complications according to the site of origin as reported in larger series. AFT: anterior fascicular tachycardia; LV: left ventricular; LVOT: left ventricular outflow tract; PFT: posterior fascicular tachycardia; RVOT: right ventricular outflow tract.

Site of Origin	Acute Procedural Success Rate, %	Complications, % Pericardial Effusion (40%) Thromboembolism (3%) Vascular Access Complications (8%) Coronary Arteries Injury (5%)
RVOT (60%)	97%	<1%
LVOT (10%)	94%	5%
LV summit (3%)	70%	5%
Right Ventricle intracavitary structures (14%)	93%	1%
Left Ventricle intracavitary structures (10%)	91%	9%
Mitral and Tricuspid Annular Region (5–10%)	90%	3%
Left bundle fascicles (10%)	90%	3%
Epicardial Foci (3–5%)	80%	8%

**Table 2:** Indications, merits, and limitations of imaging tests in patients with premature ventricular contractions. 3D, three-dimensional; CAD, coronary artery disease; CIEDs, cardiac implantable electronic devices; EF, ejection fraction; EMB, endomyocardial biopsy; LV, left ventricular; PCI, percutaneous coronary intervention; PVCs, premature ventricular contractions; RV, right ventricle; RVOT, right ventricular outflow tract; SCD, sudden cardiac death; VT, ventricular tachycardia.

Imaging Test	Indications	Advantages	Limitations
Echocardiogram	Potentially indicated in each patient presenting PVCs; may be omitted in asymptomatic healthy subjects with low PVC burden and no family history of SCD	- Widely available - Lack of contrast/radiation exposure - May be repeated over time - Allows precise measurement of LV EF	- Operator-dependent - Does not allow myocardial tissue characterization - Suboptimal visualization of complex 3D structures, including the RV
Cardiac magnetic resonance imaging	- PVCs arising from unusual locations - Sustained VT - Suspected structural heart disease by echocardiogram - Reduced LV EF	- Unlimited number of imaging planes - Accurate tissue characterization - Gold-standard assessment of ventricular structure and function (i.e., LV EF)	- Gating difficulties/artifacts due to PVCs - False positive detection of intramyocardial fat - Difficult detection of RV fibrosis - Patients with CIEDs - Gadolinium exposure
Computed tomographic coronary angiography	- Reduced LV EF - Symptoms indicating a possible underlying CAD - low-to-intermediate pre-test probability of CAD	- Non-invasive assessment of coronary anatomy - Limited radiation exposure - Possible identification of myocardial fibrosis	- Contrast and radiation exposure
Invasive coronary angiography	- Reduced LV EF - Symptoms indicating a possible underlying CAD - intermediate-to-high pre-test probability of CAD	- Gold-standard assessment of coronary anatomy - PCI in the same session	- Invasive test - Contrast and radiation exposure
Electroanatomical mapping	- Preliminary test for catheter ablation or EMB - Suspected arrhythmogenic cardiomyopathy	- Accurate assessment of the myocardial substrate - Enhances diagnostic yield of EMB	- Invasive test - Operator and tissue contact-dependent

## Data Availability

Not applicable.

## Abbreviations

Premature ventricular contractions (PVCs); structural heart disease (SHD); right and left ventricular outflow tracts (RV/LV-OT); sites of origin (SOO); nonsustained ventricular tachycardia (NSVT); sustained ventricular tachycardia (VT); catheter ablation (CA); ventricular arrhythmias (VAs); the aortic-mitral continuity (AMC); ventricular fibrillation (VF); cardiomyopathy (CMP); delayed afterdepolarizations (DADs); cyclic adenosine monophosphate (cAMP); protein kinase (protein kinase A, PKA); ryanodine receptor (RyR2); left bundle branch block (LBBB); tricuspid valve annulus (TVA); right bundle branch block (RBBB); right coronary cusp (RCC); left coronary cusp (LCC); mitral valve annulus (MVA); left ventricular summit (LVS); left circumflex (LCx); anterior interventricular vein (AIV); transthoracic echocardiogram (TTE); left ventricular ejection fraction (LVEF); arrhythmogenic right ventricular cardiomyopathy (ARVC); computed tomographic (CT); cardiac magnetic resonance (CMR); mitral valve (MV); tricuspid valve (TV); electroanatomical mapping (EAM); endomyocardial biopsy (EMB); major adverse cardiac events (MACE); antiarrhythmic drugs (AAD); Cardiac Arrhythmia Suppression Trial (CAST); intracardiac echocardiography (ICE).
